# Intraperitoneal Chemotherapy without Bevacizumab versus Intravenous Chemotherapy with Bevacizumab as the Frontline Adjuvant Therapy in Advanced Ovarian Cancer

**DOI:** 10.3390/cancers16193382

**Published:** 2024-10-03

**Authors:** Wan-Hua Ting, Hui-Hua Chen, Ming-Chow Wei, Hsu-Dong Sun, Sheng-Mou Hsiao

**Affiliations:** 1 Department of Obstetrics and Gynecology, Far Eastern Memorial Hospital, New Taipei 220216, Taiwanwei@mail.femh.org.tw (M.-C.W.);; 2 Department of Electrical Engineering, Yuan Ze University, Taoyuan 320315, Taiwan; 3 Graduate Institute of Medicine, Yuan Ze University, Taoyuan 320315, Taiwan; 4 Faculty of Medicine, School of Medicine, National Yang Ming Chiao Tung University, Taipei 112304, Taiwan; 5 Department of Obstetrics and Gynecology, National Taiwan University College of Medicine, National Taiwan University Hospital, Taipei 100226, Taiwan; 6 Graduate School of Biotechnology and Bioengineering, Yuan Ze University, Taoyuan 320315, Taiwan

**Keywords:** intravenous, intraperitoneal, ovarian cancer, chemotherapy, bevacizumab

## Abstract

**Simple Summary:**

There is no literature that compares the clinical outcomes between intraperitoneal chemotherapy without bevacizumab and triweekly intravenous chemotherapy plus bevacizumab. The aim of this study was to elucidate whether, in the frontline treatment of advanced ovarian, fallopian tube and primary peritoneal cancer, intraperitoneal cisplatin/paclitaxel chemotherapy without bevacizumab will confer an improved survival benefit when compared with the combination of triweekly intravenous carboplatin/paclitaxel chemotherapy and bevacizumab. We found that intraperitoneal chemotherapy without bevacizumab is associated with better survival when compared with intravenous chemotherapy with bevacizumab.

**Abstract:**

**Objectives:** To compare the clinical outcomes of intravenous carboplatin/paclitaxel chemotherapy plus bevacizumab versus intraperitoneal cisplatin/paclitaxel chemotherapy without bevacizumab as the frontline treatment in women with advanced ovarian, fallopian tube and primary peritoneal cancer. **Methods:** Between November 2012 and January 2024, medical records of all consecutive women with stage II~IV cancer treated with either frontline adjuvant intraperitoneal cisplatin/paclitaxel without bevacizumab (IP group), intravenous carboplatin/paclitaxel without bevacizumab (IV group) or intravenous carboplatin/paclitaxel with bevacizumab (IVB group) at a tertiary referral center were reviewed. **Results:** A total of 143 women (IP group, n = 57; IVB group, n = 23; IV group, n = 63) were reviewed. The IP group had greater progression-free survival compared to the IVB group (49.1 months, 95% confidence interval [CI] = 27.8 months to infinity, versus 11.9 months, 95% CI = 11.2 to 16.2 months; adjusted hazard ratio [HR] = 0.45, 95% CI = 0.24 to 0.87, *p* = 0.017). Additionally, the IP group also had a higher overall survival compared to the IVB group (not reached, 95% CI = 55.6 months to infinity, versus 38.9 months, 95% CI = 21.9 months to infinity; adjusted HR = 0.34, 95% CI = 0.15 to 0.79, *p* = 0.012). **Conclusions:** Intraperitoneal cisplatin/paclitaxel chemotherapy without bevacizumab seems to offer a survival advantage when compared with intravenous carboplatin/paclitaxel with bevacizumab in the frontline treatment of women with advanced ovarian cancer.

## 1. Introduction

Platinum-based chemotherapy is the mainstay for the treatment of advanced ovarian, fallopian tube and peritoneal cancer following cytoreductive surgery. Nevertheless, the majority of women will develop recurrent cancer and eventually succumb to the disease. Various treatment approaches, such as different dosing schedules, routes of administration and integration of maintenance therapy, have been tested in prospective trials, in an attempt to improve survival. National guidelines including those of the National Comprehensive Cancer Network (NCCN) and the European Society for Medical Oncology and European Society of Gynecological Oncology (ESMO-ESGO) have recommended genetic testing (germline and/or somatic) that may inform future options for maintenance therapy in women with ovarian cancer, but there is no consensus on the standard testing strategy [1,2,3].

Several trials (including the Gynecology Oncologic Group (GOG) 172 trial) [4] have demonstrated a clinically significant survival advantage associated with intraperitoneal (IP) platinum-based combination chemotherapy compared to intravenous platinum-based combination chemotherapy in advanced disease [4,5,6]. On the other hand, the final results of both GOG 218 and ICON 7 studies demonstrated no survival benefit with the addition of bevacizumab to carboplatin/paclitaxel chemotherapy [7,8,9,10]. The overall survival (OS) benefit was recorded only in poor-prognosis women (i.e., stage IV disease, inoperable stage III disease or suboptimally debulked (>1 cm) stage III disease in the ICON 7 trial) [10]. Based on the conclusions drawn from the GOG 172, GOG 218 and ICON 7 studies, IP chemotherapy seems to be a favorable option over intravenous chemotherapy plus bevacizumab in the frontline treatment of women with advanced disease [4,7,8,9,10]. However, the GOG 252 study reported no progression-free survival (PFS) or OS benefit with the use of IP chemotherapy when compared with intravenous chemotherapy, both combined with bevacizumab [11]. Thus, the incorporation of IP chemotherapy in the treatment of advanced ovarian cancer becomes questionable in the era of bevacizumab [12].

To the best of our knowledge, there is no literature that compares the clinical outcomes between IP cisplatin/paclitaxel chemotherapy without bevacizumab and intravenous carboplatin/paclitaxel chemotherapy plus bevacizumab. Thus, we were interested in defining the role of IP chemotherapy without bevacizumab, and we aimed to elucidate whether there was a survival difference between the IP cisplatin/paclitaxel chemotherapy without bevacizumab and the combination of intravenous carboplatin/paclitaxel chemotherapy and bevacizumab in the frontline treatment of advanced ovarian, fallopian tube and primary peritoneal cancer.

## 2. Materials and Methods

From November 2012 to January 2024, the medical records of all consecutive women aged 20 and above with International Federation of Gynecology and Obstetrics (FIGO) stage II–IV advanced ovarian, fallopian tube or primary peritoneal cancer who received debulking surgery, followed by either intravenous chemotherapy with bevacizumab (i.e., the IVB group), intravenous chemotherapy without bevacizumab (i.e., the IV group) or IP chemotherapy (i.e., the IP group) in a tertiary referral center were reviewed. Women who received neoadjuvant chemotherapy followed by interval debulking surgery were also eligible for participation. IP chemotherapy was defined as having one or more cycles of an IP regimen administered. Optimal debulking surgery was defined as having residual tumor with a maximum diameter less than 1 cm. The choice of chemotherapy was solely based on the physician’s preference. IP chemotherapy was performed by four of six physicians, and it was mainly delivered by two of the physicians (i.e., IP-preferring physicians), who are familiar with IP chemotherapy port implantation, irrespective of the amount of residual disease after primary surgery, as long as the women consented to the treatment plan. Bevacizumab was added to the intravenous chemotherapy by the rest of the physicians, if affordable, since its cost was not covered by the national health insurance. This study was approved by the Research Ethics Review Committee of the hospital.

The intravenous chemotherapy regimen was given as 175 mg/m^2^ paclitaxel and carboplatin at a dose calculated to produce an area under the curve (AUC) of 6 mg/mL per min on day 1. The dose of carboplatin was calculated with the formula of Calvert and colleagues [13]. Bevacizumab was given at a dose of 7.5 mg/kg intravenously on day 2 from cycle 2. The treatments were repeated every 3 weeks for six cycles. Women who did not show a complete response after six cycles of chemotherapy were potentially treated with an additional one or two cycles of chemotherapy. Bevacizumab was continued for 12 additional cycles or until disease progression, death, unacceptable toxic effects or patient voluntary withdrawal [8]. The IP regimen was given as 135 mg/m^2^ intravenous paclitaxel over a 3 h period on day 1, followed by 100 mg/m^2^ intraperitoneal cisplatin on day 2 and 60 mg/m^2^ intraperitoneal paclitaxel on day 8 [4,14]. For women with significantly impaired renal function (i.e., estimated glomerular filtration rate < 50 mL/min per 1.73 m^2^), carboplatin was used instead of cisplatin.

In women with measurable tumors, the treatment response was evaluated according to the World Health Organization criteria [15]. Otherwise, Rustin’s criteria were used in women with a cancer antigen-125 (CA-125) ≥ 40 IU/mL and without measurable tumors [16,17]. A complete response (CR) was defined as the disappearance of all evidence (including CA-125 and images) of the tumor for at least 4 weeks. A partial response (PR) was defined as a ≥50% reduction in the sum of the products of the perpendicular diameters of all measurable lesions when compared with baseline in two observations at least 4 weeks apart, or a ≥50% reduction in CA-125 for at least 4 weeks. Progressive disease (PD) was defined as a ≥25% increase in the sum of the products of the perpendicular diameters of all measurable lesions, the appearance of new lesions or a ≥25% increase in CA-125. Stable disease (SD) was defined as any condition not meeting any of the above criteria [15,16,17].

Disease recurrence was assessed according to the CA-125 criteria of disease progression [15,16], the appearance of abnormal radiological findings or histological proof from biopsy analyses, whichever occurred first. 

OS was calculated as the time interval from the date of surgery or the start of neoadjuvant chemotherapy to the date of death from any cause or last follow-up. PFS was defined as the time interval from the date of surgery or the start of neoadjuvant chemotherapy to clinically defined recurrence, disease progression, death from any cause or last follow-up.

Stata version 11.0 (Stata Corp, College Station, TX, USA) was used for statistical analyses. Survival curves were generated using the Kaplan–Meier method, and differences in the survival curves were calculated with the log-rank test. A *p*-value less than 0.05 was considered statistically significant. A multivariable Cox proportional-hazards model was run using all statistically significant variables (*p* < 0.05) in the univariate analysis to identify independent predictors of PFS and OS. Correlation coefficients were calculated to reveal the interactions between variables, particularly between the type of chemotherapy and other covariates. Correlation coefficients > 0.50 were considered to be strong correlations. Proportional-hazards assumption testing using Schoenfeld residuals was also applied. If the *p*-value of the global test was <0.05, then the Cox model did not meet the proportional-hazards assumption.

## 3. Results

A total of 143 consecutive women who underwent debulking surgery and adjuvant intravenous/intraperitoneal chemotherapy with/without bevacizumab (IVB group, n = 23; IV group, n = 63; IP group, n = 57) were reviewed (Table 1). There were no significant differences in the baseline characteristics between the three groups of women except the baseline performance status (Table 1). Despite two physicians being preferred to perform IP chemotherapy compared to the other four physicians (correlation coefficient = 0.538, Table 1), there was no difference in the residual disease status between the IP-preferring physician group and the other physician group (i.e., the IP-preferring physician group vs. the other physician group: no residual—22 vs. 12, optimal—29 vs. 10, suboptimal—41 vs. 29, correlation coefficient = −0.075). Seven women underwent neoadjuvant chemotherapy. One woman had R0, two women had an <1 cm residual tumor and four women had an >1 cm residual tumor after interval debulking surgery.

**Figure 1:**
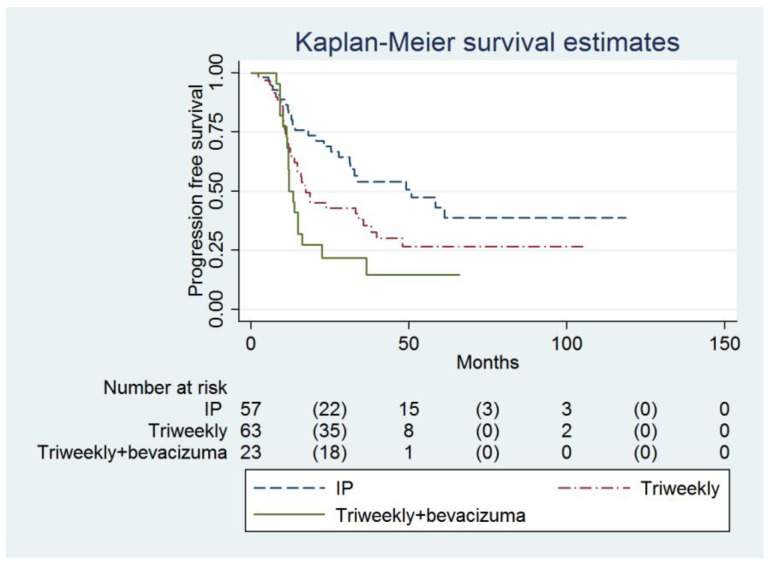
Probabilities of progression-free survival in all enrolled women (n = 143).

Multivariable analysis showed that the IP group had a higher PFS compared to the IVB group (49.1 months, 95% confidence interval [CI] = 27.8 months to infinity, versus 11.9 months, 95% CI = 11.2 to 16.2 months; adjusted hazard ratio [HR] = 0.45, 95% CI = 0.24 to 0.87, *p* = 0.017, Table 2, Figure 1). The baseline CA-125 value (adjusted HR = 1.00007, 95% CI = 1.00001 to 1.00014, *p* = 0.031) and FIGO Stage were determined to be the independent predictors of PFS (Table 2).

**Figure 2:**
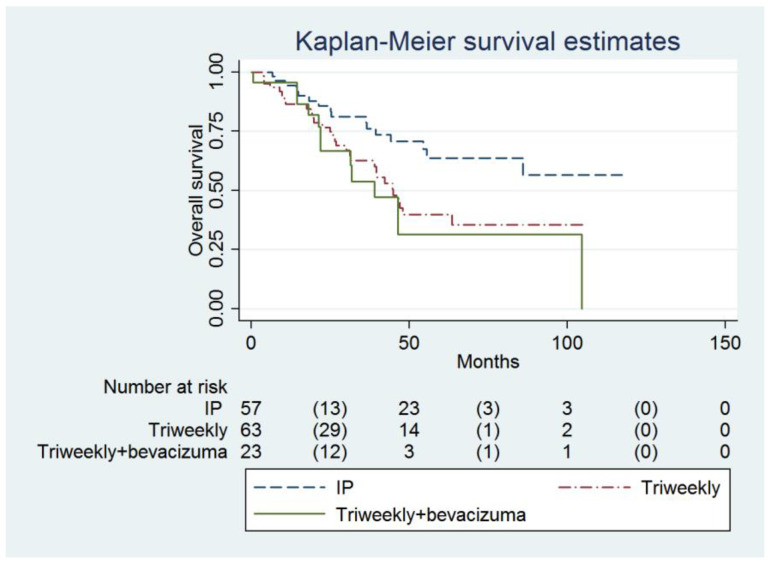
Probabilities of overall survival in all enrolled women (n = 143).

The IP group also had a higher OS compared to the IVB group (not reached, 95% CI = 55.6 months to infinity, versus 38.9 months, 95% CI = 21.9 months to infinity; adjusted HR = 0.34, 95% CI = 0.15 to 0.79, *p* = 0.012, Table 3, Figure 2). Age (adjusted HR = 1.03, 95% CI = 1.00 to 1.06, *p* = 0.022), FIGO Stage and the number of chemotherapy cycles (adjusted HR = 0.70, 95% CI = 0.58 to 0.84, *p* <0.001) were independent predictors of OS (Table 3).

A CA-125 value of ≥1176 U/mL was determined to be the optimal cut-off value for predicting disease recurrence/progression, with an AUC of 0.620 (95% CI = 0.527 to 0.712; sensitivity = 49.35%, specificity = 68.75%, Supplementary Figure S1). An age ≥ 55 years old was the optimal cut-off value for predicting death, with an AUC of 0.601 (95% CI = 0.507 to 0.695; sensitivity = 79.66%, specificity = 42.86%, Supplementary Figure S2). A number of chemotherapy cycles ≤ 5 was the optimal cut-off value for predicting death, with an AUC of 0.478 (95% CI = 0.389 to 0.567; sensitivity = 89.29%, specificity = 22.03%, Supplementary Figure S3).

## 4. Discussion

In our study, the use of IP without bevacizumab was associated with a better PFS and OS when compared with IVB. The adjusted HR of the IP was 0.45 in PFS compared to IVB (Table 2). The above result means that the risk of progression/recurrence reduced by 55% when using IP without bevacizumab as the frontline treatment compared to IVB. Additionally, compared to IVB, the adjusted HR of the IP was 0.34 in OS (Table 3). The above finding means that the risk of death reduced by 66% when using IP without bevacizumab as the frontline treatment compared to IVB. Similarly, a phase II trial also found that women with newly diagnosed ovarian cancer who received intraperitoneal chemotherapy without bevacizumab seemed to have a superior OS compared to those who received intraperitoneal chemotherapy plus bevacizumab (median OS: 79.7 versus 68 months) [18]. In addition, bevacizumab was reported to have no survival benefit in the GOG 218 and ICON 7 studies [9,10]. In the most recent Japanese phase II/III iPOCC trial, intraperitoneal carboplatin in combination with dose-dense intravenous paclitaxel without bevacizumab improved PFS versus an intravenous regimen in women with advanced ovarian cancer, regardless of the residual tumor size after initial surgery [19]. Taken together with our study, IP without bevacizumab seems to have a superior survival advantage as a frontline adjuvant treatment for advanced ovarian cancer compared to an intravenous regimen plus bevacizumab.

IP treatment provides a pharmacological advantage by directly exposing the tumor to a greater concentration of chemotherapeutic drugs [20], whereas bevacizumab causes a reduction in tumor vascularization and angiogenesis [7,8]. The reason why our IP group had a better survival benefit is unknown (Table 2 and Table 3). However, antiangiogenic therapy could prune tumor vessels excessively, rather than normalizing them, and thus decrease the delivery of chemotherapeutic agents [21]. Tumor angiogenesis can rapidly rebound when discontinuing vascular endothelial growth factor inhibition [22,23]. The above phenomenon might be a feasible explanation to justify our finding of a superior survival benefit in the IP group when compared with the IVB group.

An increased number of IP or intravenous chemotherapy cycles was a predictor of better OS (adjusted HR = 0.70, *p* < 0.001, Table 3). From our ROC analysis, we found that a number of IP or intravenous chemotherapy cycles ≤ 5 was the optimum cut-off value to predict death (Supplementary Figure S3). That is, an increase in the number of chemotherapy cycles seems to have a positive effect on the OS, and at least five cycles of chemotherapy was suggested. Similarly, both Yen et al. and Ting et al. reported that at least five cycles were needed to effectively prolong survival in women treated with IP [14,24]. 

In our study, advanced age was a predictor of poor OS (adjusted HR = 1.03, *p* = 0.026, Table 3), and the age cut-off was ≥55 years when using ROC analysis to predict death (Supplementary Figure S2). Kim et al. concluded in his study that an age cut-off of 66 years may be the prognostic indicator and the optimal starting point for a comprehensive geriatric assessment of ovarian cancer patients, especially in the serous histological subtype [25]. An older age at diagnosis was reported to have an adverse effect on the survival outcome in ovarian cancer patients, partly due to the greater likelihood of elderly patients having an advanced stage and higher-grade disease, but tumor biology differences in various age groups are also a potential explanation [26]. 

A higher baseline CA-125 level was associated with a poor PFS (adjusted HR = 1.00007, *p* = 0.031, cut-off value ≥ 1176 U/mL to predict recurrence/progression, Table 2). In the past, attempts have been made to determine an accurate CA-125 cut-off value to predict the optimal primary surgical cytoreduction, since the extent of surgery is predictive of cancer-related survival. It was proposed that higher preoperative serum CA125 values are directly related to a larger tumor burden. Baseline CA-125 has been used as a predictor of optimal cytoreduction [27,28,29], and 313.6 [27] and 500 U/mL [28] were identified to be optimal cut-off values for predicting the optimal cytoreduction. Nonetheless, Chi et al. reported that there was no identified threshold CA125 to predict the optimal cytoreduction [29]. In our study, a serum CA-125 value ≥ 1176 U/mL was the optimal cut-off level for predicting disease recurrence/progression. Similarly, a Gynecologic Oncology Group study also found that shorter PFS was observed with increasing CA-125 [30]. However, the role of CA-125 in predicting disease recurrence/progression seems to be limited due to its low AUC (AUC = 0.620 in our study). In any case, identifying a universal threshold serum CA-125 value to predict optimal cytoreduction or disease recurrence may not be possible because physicians vary in their surgical aggressiveness [28].

We acknowledge that the clinical evidence of this study is limited due to its retrospective nature, limited sample size, nonrandomized nature and the choice of chemotherapy based on the physician’s preference. In addition, bevacizumab 7.5 mg/kg was used in this study, instead of 15 mg/kg. Furthermore, the 95%CI for PFS of the IP group was wide (“27.8 months to infinity”) (Table 1), which indicates uncertainty in the estimate. Further follow-up with a larger sample size of women receiving IP treatment might improve the estimate’s precision. Despite the iPOCC trial revealing that intraperitoneal therapy improved PFS regardless of the residual tumor size after initial surgery [19], the finding that our patients underwent intraperitoneal chemotherapy irrespective of the amount of residual disease after primary surgery might stand as one of the limitations of this study. 

Furthermore, in this study, some baseline characteristics such as the extent of cytoreduction and performance status were not well-balanced between groups. In addition, some patients received neoadjuvant chemotherapy, and bevacizumab was added to the intravenous chemotherapy only if affordable. However, we used multivariable analysis in an attempt to minimize the bias. 

## 5. Conclusions

In conclusion, intraperitoneal chemotherapy with cisplatin/paclitaxel seems to be associated with a better survival benefit compared to intravenous carboplatin/paclitaxel chemotherapy with bevacizumab in the frontline treatment of women with advanced ovarian, fallopian tube or primary peritoneal cancer.

## Figures and Tables

**Table 1:** Baseline characteristics of women with advanced ovarian, fallopian tube or primary peritoneal cancer (n = 143).

Variable	Intravenous + Bevacizumab (n = 23)	Intravenous (n = 63)	Intraperitoneal (n = 57)	*p* ^a^
Age (years)	54.7 ± 12.5	60.0 ± 10.2	58.2 ± 9.9	0.108
Body mass index (kg/m^2^)	23.4 ± 2.4	24.2 ± 4.1	24.9 ± 5.3	0.762
Baseline CA-125 (U/mL)	3012 ± 3994	1697 ± 2602	2058 ± 3070	0.241
ECOG Score				
0	6 (26)	21 (33)	3 (5)	0.001
1	11 (48)	28 (44)	41 (72)	
2	6 (26)	12 (19)	13 (23)	
3	0 (0)	2 (3)	0 (0)	
FIGO Stage				
2	1 (4)	9 (14)	6 (11)	0.341
3	12 (52)	40 (63)	37 (65)	
4	10 (43)	14 (22)	14 (25)	
Site				
Ovary	22 (96)	58 (92)	52 (83)	0.312
Fallopian tube	1 (4)	0 (0)	2 (4)	
Peritoneum	0 (0)	5 (8)	3 (5)	
Histologic subtype				
Serous	13 (57)	45 (71)	41 (72)	0.622
Clear cell	4 (17)	7 (11)	5 (9)	
Others	6 (26)	11 (17)	11 (19)	
Cell grade				
1	0 (0)	2 (3)	3 (5)	0.660
2	1 (4)	4 (6)	1 (2)	
3	22 (96)	55 (87)	51 (89)	
Not available	0 (0)	2 (3)	2 (4)	
Tumor HRD status				
Negative HRD	4 (17)	5 (8)	10 (18)	0.594
Positive HRD and no tumor BRCA mutation	1 (4)	2 (3)	5 (9)	
Tumor BRCA mutation	0 (0)	1 (2)	7 (12)	
Unknown	18 (78)	55 (87)	33 (57)	
Neoadjuvant chemotherapy	1 (4)	5 (8)	1 (2)	0.325
Debulking surgery				
No residual (R0)	5 (22)	13 (21)	21 (37)	0.070
Optimal (residual < 1 cm)	3 (13)	15 (24)	16 (28)	
Suboptimal (residual > 1 cm)	15 (65)	35 (56)	20 (35)	
Lymph node metastasis	11 (48)	21 (33)	24 (42)	0.171
Number of chemotherapy cycles	5.8 ± 1.9	6.1 ± 1.5	5.5 ± 1.5	0.125
PARP inhibitor				0.481
Olaparib	2 (9)	5 (8)	5 (9)	
Niraparib	2 (9)	1 (2)	1 (2)	
IP-preferring physicians (n = 2)	5 (22)	33 (52)	54 (95)	<0.001
Other physicians (n = 4)	18 (78)	30 (48)	3 (5)	
Follow-up interval	29.8 (18.6, 46.4)	30.5 (14.7, 47.1)	39.3 (14.6, 67.5)	0.181
Progression-free survival (months)	11.9 (11.2 to 16.3)	17.4 (12.8 to 35.5)	49.1 (27.8 to infinity)	0.007
Overall survival (months)	38.9 (21.9 to infinity)	46.4 (31.5 to infinity)	Not reached (55.6 to infinity)	0.018
Clinical response				
CR	13 (57)	49 (78)	45 (79)	0.232
PR	8 (35)	8 (13)	9 (16)	
SD	1 (4)	1 (2)	1 (2)	
PD	1 (4)	4 (6)	2 (4)	

Values are expressed as mean ± standard deviation, number (percentage), median (25–75 interquartile range) or median (95% confidence interval). BMI = body mass index, CA-125 = cancer antigen 125, CR = complete response, ECOG = Eastern Cooperative Oncology Group, FIGO = The International Federation of Gynecology and Obstetrics, HRD = homologous-recombination deficiency, IP = intraperitoneal, PARP = poly (adenosine diphosphate-ribose) polymerase, PD = progressive disease, PR = partial response, SD = stable disease. ^a^ By Wilcoxon rank-sum test, chi-square test, Fisher’s exact test, ANOVA test or log-rank test.

**Table 2:** Cox proportional-hazards model to predict progression-free survival (n = 143).

Variable	Hazard Ratio	Univariate 95% CI	*p* ^a^	Hazard Ratio	Multivariable 95% CI	*p* ^b^
Regimen						
Intravenous + bevacizumab	1.00	-	-	1.00	-	-
Intravenous	0.60	0.34 to 1.07	0.087	0.79	0.43 to 1.44	0.432
Intraperitoneal	0.38	0.21 to 0.71	0.002	0.45	0.24 to 0.87	0.017
Age (years)	1.007	0.986 to 1.030	0.505	-	-	-
Body mass index (kg/m^2^)	0.960	0.915 to 1.007	0.093	-	-	-
Baseline CA-125 (U/mL)	1.00008	1.00003 to 1.00014	0.006	1.00007	1.00001 to 100,014	0.031
ECOG Score						
0	1.00	-	-	-	-	-
1	0.80	0.46 to 1.40	0.432	-	-	-
2	1.38	0.72 to 2.63	0.335	-	-	-
3	1.70	0.39 to 7.43	0.479	-	-	-
FIGO Stage						
2	1.00	-	-	1.00	-	-
3	5.96	1.85 o 19.18	0.003	5.33	1.61 to 17.68	0.006
4	8.75	2.61 to 29.33	<0.001	6.35	1.81 to 22.22	0.004
Site						
Ovary	1.00	-	-	-	-	-
Fallopian tube	0.35	0.05 to 2.54	0.300	-	-	-
Peritoneum	1.92	0.83 to 4.43	0.126	-	-	-
Histologic subtype						
Serous	1.00	-	-	-	-	-
Clear cell	1.51	0.79 to 2.89	0.218	-	-	-
Others	0.67	0.37 to 1.21	0.189	-	-	-
Cell grade						
1	1.00	-	-	-	-	-
2	1.12	0.19 to 6.68	0.905	-	-	-
3	2.15	0.53 to 8.76	0.288	-	-	-
Tumor HRD status						
Negative HRD	1.00	-	-	-	-	-
Positive HRD and no tumor BRCA mutation	1.09	0.23 to 5.14	0.911	-	-	-
Tumor BRCA mutation	0.49	0.16 to 1.52	0.217	-	-	-
Neoadjuvant chemotherapy	1.57	0.63 to 3.89	0.334	-	-	-
Debulking surgery						
No residual (R0)	1.00	-	-	1.00	-	-
Optimal (residual < 1 cm)	2.43	1.23 to 4.80	0.011	1.67	0.82 to 3.38	0.158
Suboptimal (residual > 1 cm)	3.04	1.68 to 5.51	<0.001	1.59	0.84 to 3.02	0.156
Lymph node metastasis	1.42	0.89 to 2.29	0.144	-	-	-
Number of chemotherapy cycles	1.11	0.92 to 1.34	0.289	-	-	-
PARP inhibitor						
No	1.00	-	-	-	-	-
Niraparib	4.57 × 10^15^	0 to infinity	1.000	-	-	-
Olaparib	1.31	0.60 to 2.86	0.494	-	-	-

CI = confidence interval, FIGO = The International Federation of Gynecology and Obstetrics. ^a^ Univariate Cox proportional-hazards modeling. ^b^ Multivariable Cox proportional-hazards modeling was performed using all significant variables in the univariate analysis.

**Table 3:** Cox proportional-hazards model to predict overall survival (n = 143).

Variable	Hazard Ratio	Univariate 95% CI	*p* ^a^	Hazard Ratio	Multivariable 95% CI	*p* ^b^
Regimen						
Intravenous + bevacizumab	1.00	-	-	1.00	-	-
Intravenous	0.78	0.41 to 1.51	0.469	0.79	0.38 to 1.67	0.530
Intraperitoneal	0.38	0.18 to 0.81	0.011	0.34	0.15 to 0.79	0.012
Age (years)	1.04	1.01 to 1.07	0.002	1.03	1.00 to 1.06	0.022
Body mass index (kg/m^2^)	0.97	0.91 to 1.03	0.377	-	-	-
Baseline CA-125 (U/mL)	1.00007	0.9999 to 1.00015	0.098	-	-	-
ECOG Score						
0	1.00	-	-	-	-	-
1	0.72	0.37 to 1.39	0.329	-	-	-
2	1.78	0.89 to 3.57	0.104	-	-	-
3	1.03	0.13 to 7.88	0.977	-	-	-
FIGO Stage						
2	1.00	-	-	1.00	-	-
3	11.29	1.54 to 82.73	0.017	8.68	1.15 to 65.44	0.036
4	19.95	2.68 to148.57	0.003	14.40	1.87 to 111.11	0.010
Site						
Ovary	1.00	-	-	-	-	-
Fallopian tube	4.11 × 10^−15^	0 to infinity	1.00	2.10 × 10^−20^	-	-
Peritoneum	2.94	1.33 to 6.51	0.008	1.87	0.75 to 4.66	0.180
Histologic subtype						
Serous	1.00	-	-	-	-	-
Clear cell	0.81	0.32 to 2.05	0.654	-	-	-
Others	0.66	0.34 to 1.26	0.205	-	-	-
Cell grade						
1	1.00	-	-	-	-	-
2	0.53	0.03 to 8.64	0.656	-	-	-
3	3.25	0.45 to 23.54	0.243	-	-	-
Tumor HRD status						
Negative HRD	1.00	-	-	-	-	-
Positive HRD and no tumor BRCA mutation	2.48	0.24 to 25.74	0.446	-	-	
Tumor BRCA mutation	1.50	0.42 to 5.31	0.533	-	-	-
Neoadjuvant chemotherapy	1.35	0.49 to 3.76	0.562	-	-	-
Debulking surgery						
No residual (R0)	1.00	-	-	1.00	-	-
Optimal (residual < 1 cm)	2.32	1.02 to 5.32	0.046	2.10	0.89 to 4.96	0.091
Suboptimal (residual > 1 cm)	3.47	1.71 to 7.01	0.001	2.08	0.97 to 4.47	0.061
Lymph node metastasis	1.54	0.89 to 2.67	0.123	-	-	-
Number of chemotherapy cycles	0.83	0.69 to 0.99	0.035	0.70	0.58 to 0.84	<0.001
PARP inhibitor						
No	1.00	-	-	-	-	-
Niraparib	1.10	0.15 to 8.00	0.922	-	-	-
Olaparib	0.77	0.24 to 2.47	0.660	-	-	-

CI = confidence interval, FIGO = The International Federation of Gynecology and Obstetrics. ^a^ Univariate Cox proportional-hazards modeling. ^b^ Multivariable Cox proportional-hazards modeling was performed using all significant variables in the univariate analysis.

## Data Availability

The data presented in this study are available on request from the corresponding author. The data are not publicly available due to ethical issues.

## Supplementary Materials

The following supporting information can be downloaded at https://www.mdpi.com/article/10.3390/cancers16193382/s1, Figure S1: The receiver operating characteristic curve for the serum CA-125 value as a predictor of disease recurrence; Figure S2: The receiver operating characteristic curve for age as a predictor of death; Figure S3: The receiver operating characteristic curve for the number of chemotherapy cycles as a predictor of death.
