# Supplementary material for: Intraperitoneal Chemotherapy without Bevacizumab versus Intravenous Chemotherapy with Bevacizumab as the Frontline Adjuvant Therapy in Advanced Ovarian Cancer
Source: Cancers (Basel). 2024 Oct 3;16(19):3382. doi: 10.3390/cancers16193382 (PMC11476050; doi:10.3390/cancers16193382)
Supplement: Supplementary file 1 [file cancers-16-03382-s001.zip › cancers-3207616-supplementary.pdf]

cancers MDPI

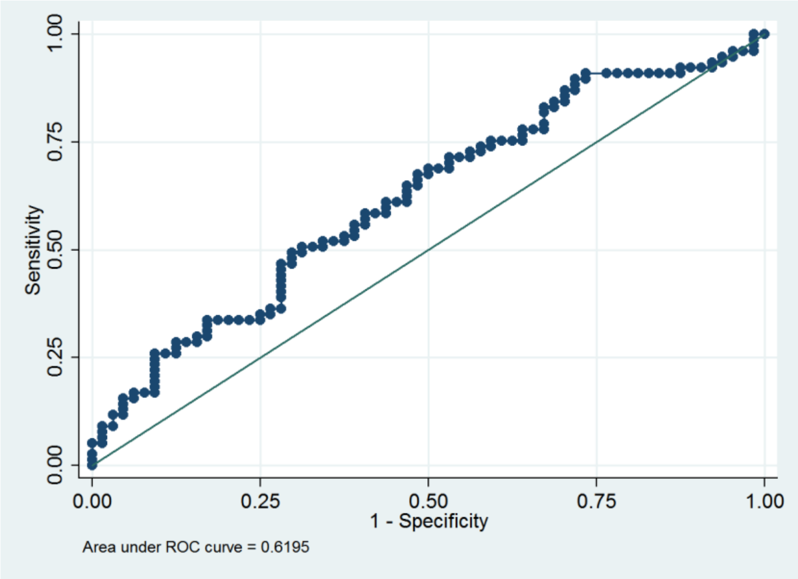

Figure S1: The receiver operating characteristic curve for serum CA-125 value as a predictor of disease recurrence.

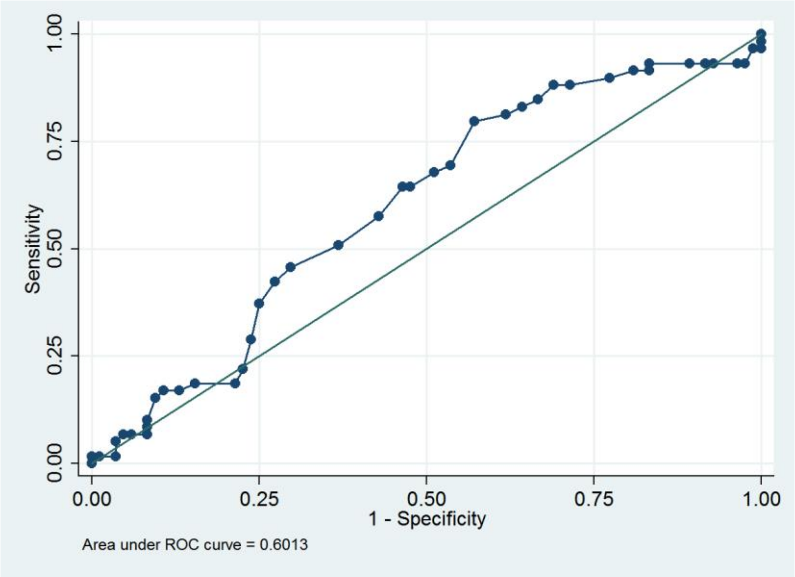

Figure S2: The receiver operating characteristic curve for age as a predictor of death.

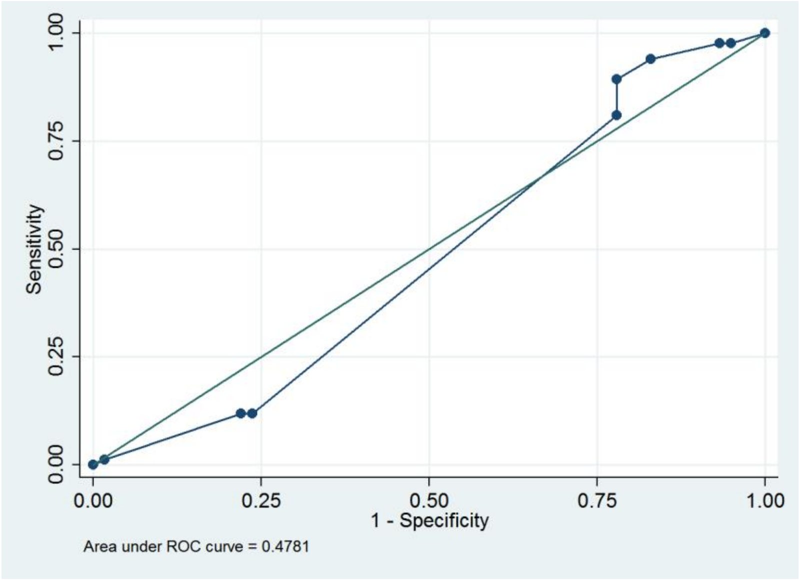

Figure S3: The receiver operating characteristic curve for the number of chemotherapy cycles as a predictor of death.
